# Prognostic implications of immunohistochemically detected YKL-40 expression in breast cancer

**DOI:** 10.1186/1477-7819-5-17

**Published:** 2007-02-07

**Authors:** Steve H Kim, Kasturi Das, Shahla Noreen, Frederick Coffman, Meera Hameed

**Affiliations:** 1 Department of Surgery, New Jersey Medical School/University of Medicine and Dentistry of New Jersey, Newark, NJ, USA; 2 Department of Pathology, New Jersey Medical School/University of Medicine and Dentistry of New Jersey, Newark, NJ, USA

## Abstract

**Background:**

YKL-40 has been implicated as a mediator of collagen synthesis and extracellular matrix re-modeling as well as mitogenesis. Elevated serum levels of YKL-40 have been associated with worse survival in a variety of malignancies including breast cancer. We wished to determine if immunohistochemically detected expression had prognostic implications in breast cancer.

**Methods:**

A prospectively collected database of breast cancer patients treated at the University Hospital of Newark was used for analysis. Immunohistochemistry was performed on archived tumor tissue from 109 patients for whom full clinical information and follow up was available.

**Results:**

YKL-40 expression was noted in 37 patients (34%). YKL-40 immunoreactivity significantly correlated with larger tumor size, poorer tumor differentiation, and a greater likelihood of being estrogen and/or progesterone receptor negative. No significant correlation was demonstrated between YKL-40 status and nodal stage. At a mean follow up of 3.2 years, disease-free survival was significantly worse in the subset of patients whose tumors demonstrated YKL-40 expression compared to the non-expressors. In multivariate analysis, YKL-40 status was independent of T-stage and N-stage in predicting disease recurrence.

**Conclusion:**

Immunoreactivity for YKL-40 was a significant predictor of breast cancer relapse in this subset of patients. This was independent of T or N-stage and suggests that tumor immunohistochemistry for this protein may be a valuable prognostic marker in breast cancer.

## Background

YKL-40 was first discovered as a 40 kDa protein secreted by the MG63 human osteosarcoma cell line [1]. It was subsequently found to belong to a family of "Chitinase-Like Proteins" (CLP), named for their structural similarity to bacterial chitinases, although lacking their characteristic enzymatic activity. The family is notable for being highly phylogenetically conserved, thus not surprisingly, some of its members have been found to be fundamental mediators of collagen synthesis and extracellular matrix re-modeling as well as mitogenesis [2]. Although the unique functions of YKL-40 are unknown, its putative involvement in human disease seems to be protean. It has been implicated as playing a role in such diverse processes as bacterial sepsis, rheumatoid arthritis, inflammatory bowel disease, cirrhosis, and cancer [3-7]. Serum levels of YKL-40 have been found to be elevated in a number of different human malignancies including those of breast, ovarian, colon, CNS, bone, and skin origin; this finding has invariably been associated with poor prognosis [8-21]. The sheer diversity of these cancers suggests that this protein may play a fundamental role in the neoplastic process.

Breast cancer afflicts approximately 200,000 women per year in the United States and is the second leading cause of cancer death in this country [22]. Traditionally, primary tumor size and presence or absence of axillary lymph node metastases are considered the most important factors determining prognosis. Other factors such as degree of tumor differentiation and expression of estrogen and progesterone receptors also have prognostic import, although they are not usually used in clinical staging. In the past decade, numerous studies have reported that amplification/overexpression of the transmembrane protein kinase Her2/*neu*, may be associated with a more aggressive phenotype [23]. Although controversy persists as to this finding's true prognostic importance [24], this issue has largely been rendered moot by the recent demonstration of therapeutic efficacy of a monoclonal antibody directed against Her2 in patients with Her2-positive breast cancers [25-27]. The relatively new technology of microarray analysis [28,29] may offer further insight into new molecular prognostic markers/therapeutic targets, as it is becoming clear that this is the next evolutionary leap in cancer treatment. YKL-40 holds great promise in this regard; this assertion is supported by the finding that elevated levels of this protein are associated with poor outcome in women with invasive breast cancer [11,12,14] and ovarian cancer [15]. Heretofore, these studies have been mainly done with ELISA of patient sera. A previous immunohistochemical survey of breast cancer *tissue *demonstrated expression in over 70% of the samples, but the clinical significance of this finding was not reported [30]. In the present study, we also wished to determine the expression pattern of YKL-40 in breast cancer tissue, and further ascertain whether this had prognostic implications similar to what was found with data from patient sera.

## Materials and methods

Between May 1999 and June 2006, we prospectively collected and collated data on 265 women who underwent operative therapy for breast cancer at University Hospital in Newark. Of these, 215 patients had invasive disease (81%) and 50 patients (19%) had pure ductal-carcinoma-in-situ (DCIS). Of the patients with invasive disease, we had full clinical follow-up, tissue availability, and YKL-40 immunohistochemical data on 109 patients. These patients made up the cohort for the present study. Institutional Review Board approval was obtained via Protocol # 0120030318.

### Patient and tumor characteristics

Median age for the 109 patients in our study was 52 years. The study group was predominantly Black (N = 65, 60%) and Hispanic (N = 35, 32%), reflecting the nature of our patient population. The remainder of the cohort was Caucasian (N = 7, 6%), Asian (N = 1, 1%), and Arabic (N = 1, 1%). Breast conserving surgery was performed in 51 patients (47%), while 42 had mastectomy (38%) and 16 underwent mastectomy with immediate reconstruction (15%). Sentinel node staging was performed in 76 patients (70%). Sixteen patients were treated with neoadjuvant chemotherapy prior to surgery. Most patients (83%) were T1 or T2 with median tumor size being 2.5 cm. The majority of tumors (57%) were either poorly or moderately differentiated (degree of tumor differentiation was determined by Bloom-Richardson grade whenever possible [31]). Over half the cohort (54%) presented with node-positive disease. The median number of positive nodes for node-positive patients was 2. The median number of nodes harvested for patients undergoing complete axillary lymphadenectomy was 21. Sixty-seven patients (62%) were estrogen-receptor positive by immunohistochemistry. Her2 status was documented with a combination of immunohistochemistry and fluorescence in-situ hybridization analysis (FISH). Tumors that were strongly positive by immuno-staining were classified as positive without further analysis; if the immunohistochemical results were weak or equivocal, FISH was performed. Using this algorithm, Her2 overexpression was noted in 29 patients (27%). Patient and tumor characteristics and how they relate to YKL-40 status are summarized in Table 1. Mean follow-up was 3.0 years. Survival was analyzed via the method of Kaplan-Meier [32]. Statistical significance was determined by chi-square analysis, log rank testing, and the Cox regression model.

**Table 1:** YKL-40 immunoreactivity and breast cancer prognostic factors

**Factor**	**YKL-40 detected**	**YKL-40 not detected**	**p**
Mean/median tumor size (range)	4.0 cm/3.0 cm (0 – 20.0)	2.7 cm/2.3 cm (0 – 16.0)	<.05
Tumor differentiation*, N (%)			<.001
• Well	1 (2)	17 (24)	
• Moderate	8 (22)	25 (35)	
• Poor	20 (54)	9 (12)	
• Unknown	8 (22)	21 (29)	
ER status*, N (%)			<.01
• Negative	21 (57)	19 (27)	
• Positive	15 (41)	52 (72)	
• Unknown/Not done	1 (2)	1 (1)	
PR status*, N (%)			<.01
• Negative	26 (71)	25 (35)	
• Positive	10 (27)	46 (64)	
• Unknown/Not done	1 (2)	1 (1)	
Nodal status, N (%)			NS
• Negative	18 (49)	32 (44)	
• Positive	19 (51)	40 (56)	
Mean/median # of positive nodes (Node-positive patients only)	4.8/2.0 (1 – 22)	3.1/2.0 (1 – 17)	NS
Her2*, N (%)			NS
• Negative	27 (73)	47 (65)	
• Positive	8 (22)	21 (29)	
• Unknown/Not done	2 (5)	4 (6)	
T stage, N (%)			NS
• T1	10 (27)	33 (46)	
• T2	17 (46)	30 (42)	
• T3	4 (11)	5 (7)	
• T4	6 (16)	4 (5)	
N stage, N (%)			NS
• N0	18 (49)	33 (46)	
• N1	11 (30)	30 (41)	
• N2	7 (19)	7 (10)	
• N3	1 (2)	2 (3)	
AJCC stage, N (%)			NS
• I	6 (16)	16 (22)	
• IIA	11 (30)	28 (39)	
• IIB	6 (16)	15 (21)	
• IIIA	7 (19)	7 (10)	
• IIIB	5 (14)	4 (6)	
• IIIC	2 (5)	1 (1)	
• IV	0 (0)	1 (1)	

* For tumors in which this data was known. Statistical analysis was performed after excluding non-informative cases.

### Immunohistochemistry

Representative tumor blocks were pulled and histology of all cases was reviewed (MH). Almost all the cases were from surgical excision specimens Immunohistochemistry was performed as follows. A conventional peroxidase staining technique was performed using rabbit polyclonal YKL-40 antibody (Quidel^® ^Corporation, Santa Clara, CA). Briefly, the slides were deparaffinized and endogenous peroxidase was quenched with 3% hydrogen peroxide. Antigen retrieval was performed using Target Retrieval Solution (DAKO Cytomation^®^) in a steamer for 40 minutes, and then placed into TBS (tris-buffered saline) for at least 5 minutes. The DAKO auto-stainer was used for the following steps: after applying 100 microliters of primary antibody (YKL-40, at original concentration of 1 mg/ml used at 1:160 dilution) for 15 minutes, Envision + Rabbit kit HRP-labeled polymer (DAKO Cytomation^®^) was used for the secondary antibody and incubated for 30 minutes. The final detection was performed by using DAB as the chromogen with hematoxylin (3%) used as the counterstain. A glioblastoma multiforme was used as positive control (immunoreactivity is noted in the tumor cells)[10]. For every case, a negative control without a primary antibody was used. In addition, fibroblasts and blood vessels were used as internal negative controls. Positive staining was recognized as brown color in the cytoplasm of tumor cells.

## Results

**Figure 1:**
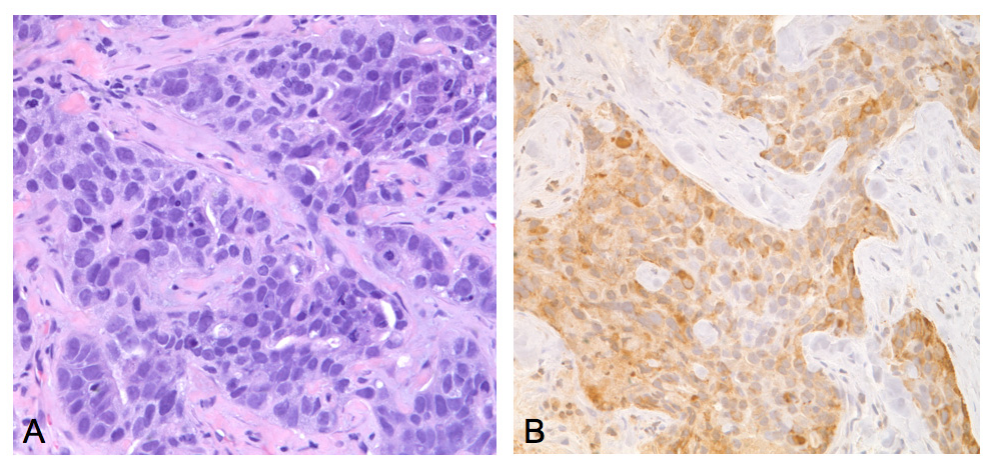
**A**: Poorly differentiated ductal adenocarcinoma, H&E stain (400×). **B**: Same patient showing YKL-40 immunoreactivity within the cytoplasm of tumor cells (400×).

All immunohistochemical evaluation and scoring was performed by one of the authors (MH). Evaluation was always performed in an area of tumor where malignant cells were the most abundant. YKL-40 immunoreactivity was recognized as brown staining within cells, and was localized mainly to the cytoplasm of tumor cells (Figure 1). The staining intensity was more or less equal between the tumors and thus was not included as a scoring criterion. The number of reactive cells per specimen was variable, ranging from only a few per slide to more diffuse staining in focal areas of the tumor. No case, however, demonstrated immunoreactivity in *all *tumor cells, consistent with previous reports of expression in cancer tissues [10,30]. We arbitrarily stratified these results on a scale of 0–2: a score of 0 denoted no detectable expression, 1 denoted "weak" expression (≤10 immunoreactive cells/tumor), and 2 denoted "strong" expression (>10 immunoreactive cells/tumor). A score of 1 or 2 was considered a "positive" result. Using these criteria, YKL-40-positive tumors were found in 37 patients (34%). Among the positive cases, 28/37 (76%) were considered "strong" expressors (score of 2).

We subsequently correlated the experimental findings with the clinical characteristics of our patient cohort. No significant difference was noted in the mean age of the patient subset with tumors in which YKL-40 was detected vs. the control group (52.3 years vs. 53 years, respectively). Furthermore, incidence of expression was not significantly different between invasive ductal vs lobular cancers. In contrast, several primary tumor factors associated with poor prognosis were found to correlate significantly with YKL-40 immunoreactivity (Table 1). Mean size of cancers in which the antigen was detected was 4.0 cm compared to 2.7 cm for those in which it was not (p < .05). Furthermore, the incidence of YKL-40 detection notably increased with worsening tumor differentiation (p < .001). Nearly 70% of YKL-40-positive tumors were poorly differentiated compared to only 18% of controls. The former group was also significantly more likely to be estrogen and progesterone receptor negative (p < .01). Factors demonstrating no significant correlation to YKL-40 status included presence or absence of axillary lymph node metastases, nodal stage (N0 – N3), or number of positive nodes in patients with node-positive disease. There was also no association between YKL-40 and Her2 immunoreactivity. These findings did not change when the weak YKL-40 expressing tumors (N = 9) were classified as "negative."

**Figure 2:**
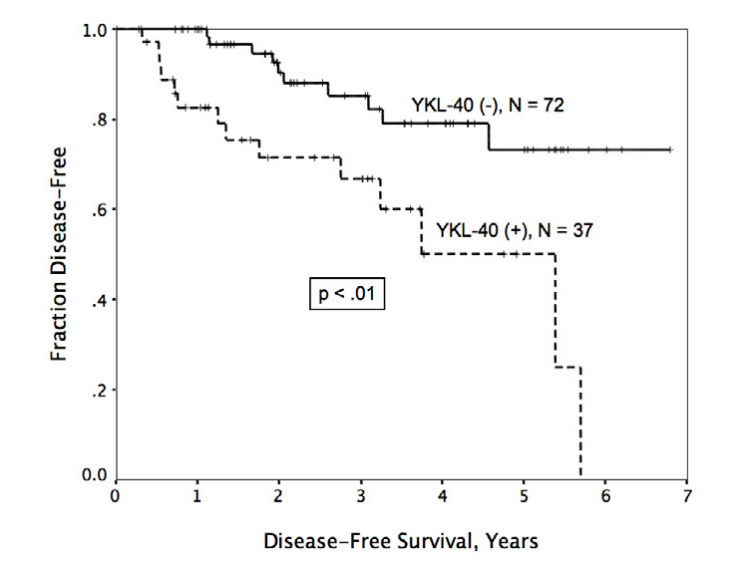
Disease-free survival was significantly worse in patients with tumors that demonstrated YKL-40 immunoreactivity.

At a mean follow up of 3.2 years (median 2.7 years, range 0.4 – 7.3 years), actuarial 5-year disease-free survival (DFS) for the entire cohort was 66%. Factors that significantly predicted poor outcome on univariate analysis included primary tumor size, nodal stage, and immunohistochemically detectable YKL-40 expression. Degree of tumor differentiation was of borderline significance (p = .05). ER, PR, and Her2 status were not predictive of disease relapse on this cohort, perhaps due to the relatively limited follow up. For patients with YKL-40-positive tumors, median DFS was only 3.8 years (patients with non-expressing tumors had not yet reached median, Figure 2). Significant univariate factors associated with poor DFS were then examined by multivariate analysis using stepwise forward Cox regression, and demonstrated that YKL-40 status was independent of tumor size and nodal stage in predicting disease recurrence. Of note, although the numbers are small, no difference in survival was noted between patients having tumors with strong YKL-40 expression (N = 28) and those having tumors with weak expression (N = 9).

## Discussion

In our study, we found that expression of YKL-40 was detected in about one-third of archived breast cancer tissue specimens surveyed using immunohistochemical analysis. We noted cytoplasmic localization of the antigen within tumor cells, a finding consistent with that reported in a similar study of glioblastomas [10] and a previous survey of breast cancers [30]. This seems consistent with the fact that YKL-40 is a secreted protein and has been detected in sera of cancer patients. Although the number of immunoreactive cells per specimen was variable, this tumor-to-tumor variability in YKL-40 expression was not associated with any difference in its prognostic efficacy, that is, cancers with only sparse expression (score 1) associated with outcome that was similar to cancers with more diffuse expression (score 2). Our results differ somewhat from that of Roslind et al [30] in that we found a much smaller fraction of breast cancer specimens in which YKL-40 was detected (71% vs. 34%). This difference may simply be attributable to methodological discrepancy, however, the latter figure seems to be more consistent with the serum data in which 19–30% of breast cancer patients were found to have elevated levels of the antigen [11,14].

Clinical correlation in our study showed that YKL-40 antigen detection was associated with larger tumor size, poorer tumor differentiation, and a higher likelihood of estrogen and progesterone receptor negativity. Furthermore, YKL-40 immunoreactivity was a predictor of poor disease-free survival in both univariate and multivariate analysis, being independent of both T- and N-stage. These results are in concordance with the serum data where high YKL-40 levels were predictive of worse outcome that also was independent of more traditional prognostic indices such as T and N stage [11,12,14]. There are, however, some notable differences between the serum studies and our results. The study by Johansen and colleagues showed a significant correlation between high serum YKL-40 level and node positivity, but no difference with intrinsic primary tumor factors such as size, grade (degree of differentiation), and estrogen receptor positivity [11]. Our results were essentially contrary: we found no statistical correlation with nodal disease however, there was a high degree of association with the above negative prognostic indices characteristic of the primary tumor (Table 1). These findings may indicate that the serum and immunohistochemical data, although both prognostically significant in determining outcome, may give different information. The immunohistochemical findings may be more strongly associated with intrinsic biology of the primary tumor, while the serum data may be more informative of nodal and distant metastasis [11,12,14]. Unquestionably, these two clinical phenomena are intimately related, so it will be useful in the future to clinically correlate the results of *combined *serum and immunohistochemical data from the same patients.

Our report does have some obvious limitations. The cohort size is relatively small and predominantly made up of women from ethnic minorities. The majority were Black females, a group that some have suggested may have an inherently worse breast cancer survivorship [33,34]. Furthermore, because of the relative immaturity of our database, the length of follow-up was somewhat limited. Clearly, the results will require confirmation using greater patient numbers, longer periods of observation, as well as a patient population that is more reflective of the entire ethnic spectrum of women afflicted with breast cancer. However, despite these caveats, we feel that the findings are notable, and lay fertile ground for future studies of YKL-40 and its role in breast cancer progression.

## Conclusion

Immunoreactivity for YKL-40 was a significant predictor of breast cancer relapse in this subset of patients. This was independent of T or N-stage and suggests that tumor immunohistochemistry for this protein may be a valuable prognostic marker in breast cancer

## Competing interests

The author(s) declare that they have no competing interests.

## Authors' contributions

**SK **contributed to the conception of the project, collection and interpretation of the data, and drafting, revising, and giving final approval of the manuscript.

**KD **contributed to collection and interpretation of the data, and revising and giving final approval of the manuscript.

**SN **contributed to collection and interpretation of the data, and revising and giving final approval of the manuscript.

**FC **contributed to the conception of the project, and revising and giving final approval of the manuscript.

**MH **contributed to the conception of the project, collection and interpretation of the data, and revising, and giving final approval of the manuscript.

All authors read and approved final manuscript
